# 1-(4-{[(1,3,3-Tri­methyl­indolin-2-yl­idene)meth­yl]diazen­yl}phen­yl)ethanone

**DOI:** 10.1107/S1600536813024124

**Published:** 2013-09-04

**Authors:** Graeme J. Gainsford, Mohamed Ashraf, M. Delower H. Bhuiyan, Andrew J. Kay

**Affiliations:** a Callaghan Innovation Research Limited, PO Box 31-310, Lower Hutt, New Zealand

## Abstract

The title compound, C_20_H_21_N_3_O, has crystallographic mirror symmetry with all non-H atoms apart from the methyl C atom of the CMe_2_ group lying on the mirror plane. Mol­ecules are linked into planar sheets parallel to (010) by phen­yl–azo C—H⋯N and phen­yl–ethanone C—H⋯O inter­actions. Methyl C—H⋯π inter­actions provide crosslinking between the planes.

## Related literature

For general background to NLO chromophores, see: Dalton *et al.* (2011 ▶); Marder *et al.* (1994 ▶); Cheng *et al.* (1991 ▶); Mashraqui *et al.* (2004 ▶); Moylan *et al.* (1993 ▶); Zhang *et al.* (1997 ▶); Prim *et al.* (1994 ▶). For related structures, see: Odabasoglu *et al.* (2005 ▶); Simunek *et al.* (2003 ▶); Bhuiyan *et al.* (2011 ▶); Ashraf *et al.* (2013 ▶). For analysis of the structures, see: Spek (2009 ▶); Bernstein *et al.* (1995 ▶). For a description of the Cambridge Structural Database, see: Allen (2002 ▶).
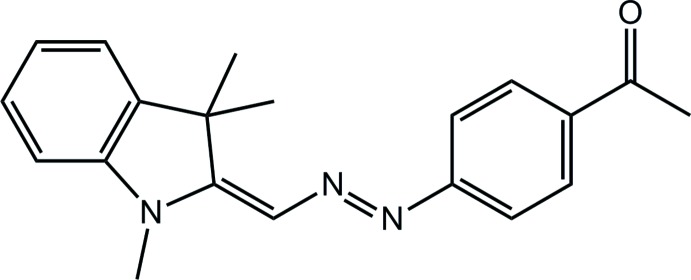


## Experimental

### 

#### Crystal data

C_20_H_21_N_3_O
*M* *_r_* = 319.40
Monoclinic, 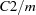

*a* = 14.8688 (2) Å
*b* = 6.89500 (12) Å
*c* = 16.3546 (3) Å
β = 99.5834 (16)°
*V* = 1653.27 (5) Å^3^
*Z* = 4
Cu *K*α radiation
μ = 0.64 mm^−1^
*T* = 120 K
0.19 × 0.15 × 0.09 mm

#### Data collection

Agilent SuperNova (Dual, Cu at zero, Atlas) diffractometer
Absorption correction: gaussian (*CrysAlis PRO*; Agilent, 2013 ▶) *T*_min_ = 0.804, *T*_max_ = 1.000
9379 measured reflections
1803 independent reflections
1634 reflections with *I* > 2σ(*I*)
*R*_int_ = 0.026

#### Refinement

*R*[*F* ^2^ > 2σ(*F* ^2^)] = 0.036
*wR*(*F* ^2^) = 0.098
*S* = 1.07
1803 reflections
153 parameters
H atoms treated by a mixture of independent and constrained refinement
Δρ_max_ = 0.27 e Å^−3^
Δρ_min_ = −0.21 e Å^−3^

### 

Data collection: *CrysAlis PRO* (Agilent, 2013 ▶); cell refinement: *CrysAlis PRO*; data reduction: *CrysAlis PRO*; program(s) used to solve structure: *SHELXS97* (Sheldrick, 2008 ▶); program(s) used to refine structure: *SHELXL2012* (Sheldrick, 2012 ▶); molecular graphics: *ORTEP-3 for Windows* (Farrugia, 2012 ▶) and *Mercury* (Macrae *et al.*, 2006 ▶); software used to prepare material for publication: *SHELXL2012*, *PLATON* (Spek, 2009 ▶) and *Mercury*.

## Supplementary Material

Crystal structure: contains datablock(s) global, I. DOI: 10.1107/S1600536813024124/tk5249sup1.cif

Structure factors: contains datablock(s) I. DOI: 10.1107/S1600536813024124/tk5249Isup2.hkl

Supplementary material file. DOI: 10.1107/S1600536813024124/tk5249Isup3.cml

Additional supplementary materials:  crystallographic information; 3D view; checkCIF report

## Figures and Tables

**Table 1:** Hydrogen-bond geometry (Å, °)

*D*—H⋯*A*	*D*—H	H⋯*A*	*D*⋯*A*	*D*—H⋯*A*
C2—H2⋯O1^i^	0.95	2.57	3.5227 (19)	179
C14—H14⋯N3^ii^	0.95	2.55	3.4985 (19)	175
C11—H11*A*⋯*Cg*3^iii^	0.981 (15)	2.665 (14)	3.5230 (4)	145.8 (11)

Symmetry codes: (i) 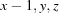; (ii) 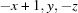; (iii) 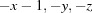.

**Fig. 1.**
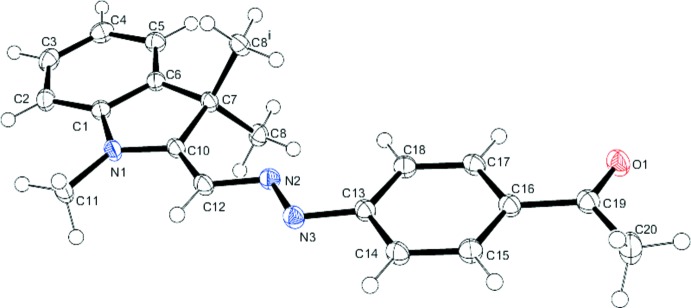
Molecular structure of title molecule; displacement ellipsoids are shown at the 50% probability level. Symmetry (i) x, -y, z.

**Fig. 2.**
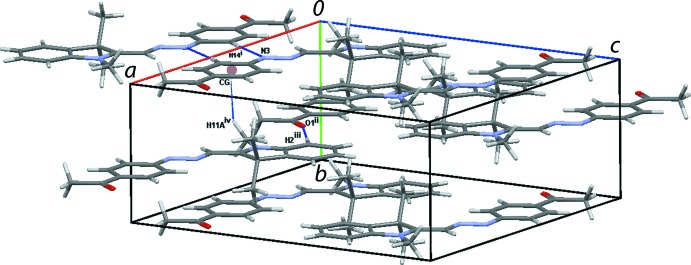
Cell packing view; one representative set of intermolecular attractive contacts (Table 1) are shown as purple dotted lines. The CG ball is the centroid of phenyl group C13···C18. Symmetry (i): 1 - x, y, -z (ii) -1/2 + x, 1/2 + y, z (iii) 1/2 + x, 1/2 + y,z (iv) 1/2 + x, 1/2 - y, z.

## supplementary crystallographic information

### 1. Comment

The synthesis of organic non-linear optical (NLO) molecules continue to be of research interest due to their potential use in optical communications, information storage, optical switching and photonic imaging and sensing (Dalton *et al.*, 2011). Dipolar donor-π-acceptor (D-π-A) type chromophores are commonly connected *via* olefins (Marder *et al.*, 1994), acetylenes (Cheng *et al.*, 1991), oxadiazole systems (Mashraqui *et al.*, 2004) and azo groups (Moylan *et al.*, 1993). However, despite the vast range of possibilities, there are some strategies for designing effective NLO materials that consistently give good results, including the incorporation of azo linkers into the conjugated interconnect. Consequently, a number of such D-azo-A systems have been investigated, with many azo-containing systems showing improved non-linear optical performance and thermal stability (Zhang *et al.*, 1997) when compared to the olefinic analogues.

Furthermore, over the past two decades, azobenzene/azoheterocycle containing polymers have been the subject of intensive research in optical switching, and digital and holographic storage applications (Prim *et al.*, 1994). Thus, they represent a useful class of compound to study as they hold promise for applications beyond just non-linear optics. Hence, there is a need to synthesize new organic NLO materials with azo linkers and study their structural, physical, thermal and optical properties. We have recently reported a range of NLO materials containing an azo linker (Ashraf *et al.*, 2013).

The asymmetric unit contains the title compound which lies on a crystallographic mirror plane (Fig. 1). The planarity of structures containing an azo linkage and indeed, the N–N bond length, varies considerably depending on the bound ring systems (Allen, 2002; CSD Version 5.34, with May 2013 updates). For example in LAQYAE (Odabasoglu *et al.*, 2005) the dihedral angles of the pendant phenyl rings being 0.31 (12) and 26.74 (14)° for the two independent molecules with N–N lengths of 1.158 (4) and 1.247 (3) Å, respectively. The closest related structure with appended phenyl and alkene chain is ULEGAT (Simunek *et al.*, 2003) with a comparable N—N length of 1.282 (2) Å, and dihedral angle 0.4 (2)°. The quality of the crystal packing & consequent diffraction data confirms that the methyl hydrogen atoms based on the in-plane carbon C11 are ordered unlike the disorder model required for the related compound 2-{3-[2-(1,3,3-trimethyl-1,3-dihydroindol-2-ylidene)propenyl] -5*H*-furan-2-ylidene}malonitrile (Bhuiyan *et al.*, 2011), hereafter FATN, which also crystallized in space group *C*2/*m*.

The planar molecules in the title compound form sheets utilizing two interactions in a similar way although with different interactions to those in FATN. Here, the phenyl(C14)–H14···N3(azo) interaction provides one of the in-plane links making the common *R*^2^_2_(8) motif (Bernstein *et al.*, 1995) parallel to ethene(C)—H···N3(cyano) *R*^2^_2_(16) interaction in FATN, aligned around a two fold axis of symmetry (Fig. 2). Likewise, a second in-plane interaction here occurs between phenyl(C2)—H and the ketone oxygen O1 described by the C(14) motif whilst in FATN a (dichloromethane)C–Cl···N(cyano) interaction performs the same role. A methylC–H···O(ketone) interaction is also found in the ULEGAT crystal packing. The planar (0 1 0) sheets are then cross-linked by two (symmetric) methyl(C11)–H11A···π(phenyl) interactions as shown in Fig. 2.

### 2. Experimental

4-Aminacetophenone (1.35 g, 10 mmol) was added to concentrated sulfuric acid (10 ml) and the reaction mixture cooled to 0–5 °C. A solution of sodium nitrite (824 mg, 12 mmol) in water (5 ml) was added slowly and the reaction stirred at 0–5 °C for 30 min. To this, was added a solution of 1,3,3-trimethyl 2-methyleneindole (1.56 g, 9 mmol) in acetic acid (20 ml); the resultant mixture was stirred for an additional 2 h and then poured into water and neutralized with aqueous sodium carbonate. The resulting oil was extracted with dichloromethane, dried (MgSO_4_) and concentrated under reduced pressure. The crude material was purified by column chromatography (silica gel, dichloromethane: hexanes 1:1) to afford the final compound (1.89 g, 66%) as a bright-red-yellow solid. X-ray quality red crystals were grown by slow evaporation of a solution of the chromophore in a chloroform and methanol mixture (1:1). *M*.pt: XXX K. ^1^H NMR (500 MHz, DMSO-d6) δ: 1.71 (6H, s, 2x CH_3_), 2.58 (3H, s, CO—CH_3_), 3.43 (3H, s, N—CH_3_), 7.05–7.12 (1H, m, ArH), 7.19 (1H, d, J 5.0 Hz, ArH), 7.30–7.35 (1H, m, ArH), 7.45 (1H, d J 5.0 Hz, ArH), 7.51 (1H, s, CH), 7.63 (2H, d, J 5.0 Hz, ArH), 8.02 (2H, d, J 10.0 Hz, ArH). ^13^C NMR (125 MHz, DMSO-d6) δ: 26.6, 28.5, 29.8, 48.0, 108.9, 120.4, 120.8, 122.0, 122.4, 127.9, 128.1, 129.6, 134.4, 138.4, 139.8, 143.8, 156.9, 164.8, 168.0, 196.8.

### 3. Refinement

All H atoms except those on C11 & C20 bound to carbon were constrained to their expected geometries (C—H 0.95–0.98 Å). The methyl-H atoms bound to atoms on the mirror plane were located on difference Fourier maps and their positions refined. All methyl- and phenyl-H atoms were refined with *U*_iso_ 1.5 & 1.2 times, respectively, that of the *U*_eq_ of their parent atom.

### Crystal data

**Table:**

C_20_H_21_N_3_O	*F*(000) = 680
*M**_r_* = 319.40	*D*_x_ = 1.283 Mg m^−^^3^
Monoclinic, *C*2/*m*	Cu *K*α radiation, λ = 1.5418 Å
*a* = 14.8688 (2) Å	Cell parameters from 4498 reflections
*b* = 6.89500 (12) Å	θ = 5.5–73.9°
*c* = 16.3546 (3) Å	µ = 0.64 mm^−^^1^
β = 99.5834 (16)°	*T* = 120 K
*V* = 1653.27 (5) Å^3^	Block, red
*Z* = 4	0.19 × 0.15 × 0.09 mm

### Data collection

**Table:**

Agilent SuperNova (Dual, Cu at zero, Atlas) diffractometer	1803 independent reflections
Radiation source: SuperNova (Cu) X-ray Source	1634 reflections with *I* > 2σ(*I*)
Mirror monochromator	*R*_int_ = 0.026
Detector resolution: 10.6501 pixels mm^-1^	θ_max_ = 73.8°, θ_min_ = 2.7°
ω scans	*h* = −17→18
Absorption correction: gaussian (*CrysAlis PRO*; Agilent, 2013)	*k* = −8→8
*T*_min_ = 0.804, *T*_max_ = 1.000	*l* = −20→19
9379 measured reflections	

### Refinement

**Table:**

Refinement on *F*^2^	0 restraints
Least-squares matrix: full	Hydrogen site location: mixed
*R*[*F*^2^ > 2σ(*F*^2^)] = 0.036	H atoms treated by a mixture of independent and constrained refinement
*wR*(*F*^2^) = 0.098	*w* = 1/[σ^2^(*F*_o_^2^) + (0.0502*P*)^2^ + 0.9765*P*] where *P* = (*F*_o_^2^ + 2*F*_c_^2^)/3
*S* = 1.07	(Δ/σ)_max_ < 0.001
1803 reflections	Δρ_max_ = 0.27 e Å^−^^3^
153 parameters	Δρ_min_ = −0.21 e Å^−^^3^

### Special details

**Table:**

Geometry. All e.s.d.'s (except the e.s.d. in the dihedral angle between two l.s. planes) are estimated using the full covariance matrix. The cell e.s.d.'s are taken into account individually in the estimation of e.s.d.'s in distances, angles and torsion angles; correlations between e.s.d.'s in cell parameters are only used when they are defined by crystal symmetry. An approximate (isotropic) treatment of cell e.s.d.'s is used for estimating e.s.d.'s involving l.s. planes.

### Fractional atomic coordinates and isotropic or equivalent isotropic displacement parameters (Å^2^)

**Table:**

	*x*	*y*	*z*	*U*_iso_*/*U*_eq_	
O1	0.93789 (7)	0.0000	0.21729 (7)	0.0256 (3)	
N1	0.24599 (8)	0.0000	0.25969 (8)	0.0192 (3)	
N2	0.46957 (8)	0.0000	0.19967 (7)	0.0186 (3)	
N3	0.50012 (8)	0.0000	0.13053 (8)	0.0204 (3)	
C1	0.22453 (9)	0.0000	0.34038 (9)	0.0187 (3)	
C2	0.13956 (10)	0.0000	0.36490 (10)	0.0227 (3)	
H2	0.0848	0.0000	0.3256	0.027*	
C3	0.13841 (11)	0.0000	0.44990 (11)	0.0255 (3)	
H3	0.0814	0.0000	0.4690	0.031*	
C4	0.21825 (11)	0.0000	0.50739 (10)	0.0245 (3)	
H4	0.2153	0.0000	0.5650	0.029*	
C5	0.30291 (10)	0.0000	0.48133 (9)	0.0216 (3)	
H5	0.3577	0.0000	0.5206	0.026*	
C6	0.30564 (9)	0.0000	0.39733 (9)	0.0185 (3)	
C7	0.38604 (9)	0.0000	0.35149 (9)	0.0177 (3)	
C8	0.44476 (7)	0.18267 (16)	0.37151 (6)	0.0217 (2)	
H8A	0.4685	0.1868	0.4311	0.033*	
H8B	0.4075	0.2981	0.3557	0.033*	
H8C	0.4958	0.1796	0.3405	0.033*	
C10	0.33759 (9)	0.0000	0.26123 (9)	0.0176 (3)	
C11	0.17867 (10)	0.0000	0.18420 (10)	0.0232 (3)	
H11A	0.1863 (9)	0.114 (2)	0.1498 (9)	0.035*	
H11B	0.1170 (14)	0.0000	0.1982 (13)	0.035*	
C12	0.37724 (10)	0.0000	0.19120 (9)	0.0191 (3)	
H12	0.3407	0.0000	0.1377	0.023*	
C13	0.59616 (10)	0.0000	0.14061 (9)	0.0188 (3)	
C14	0.63291 (10)	0.0000	0.06719 (9)	0.0215 (3)	
H14	0.5933	0.0000	0.0152	0.026*	
C15	0.72657 (10)	0.0000	0.06964 (9)	0.0222 (3)	
H15	0.7505	0.0000	0.0193	0.027*	
C16	0.78629 (10)	0.0000	0.14535 (9)	0.0196 (3)	
C17	0.74891 (10)	0.0000	0.21879 (9)	0.0201 (3)	
H17	0.7886	0.0000	0.2707	0.024*	
C18	0.65596 (10)	0.0000	0.21732 (9)	0.0208 (3)	
H18	0.6322	0.0000	0.2678	0.025*	
C19	0.88757 (10)	0.0000	0.15055 (10)	0.0214 (3)	
C20	0.92658 (11)	0.0000	0.07131 (11)	0.0316 (4)	
H20A	0.9913 (16)	0.0000	0.0818 (15)	0.047*	
H20B	0.9057 (10)	0.113 (2)	0.0369 (10)	0.047*	

### Atomic displacement parameters (Å^2^)

**Table:**

	*U*^11^	*U*^22^	*U*^33^	*U*^12^	*U*^13^	*U*^23^
O1	0.0193 (5)	0.0339 (6)	0.0220 (6)	0.000	−0.0014 (4)	0.000
N1	0.0140 (6)	0.0235 (6)	0.0189 (6)	0.000	−0.0007 (5)	0.000
N2	0.0185 (6)	0.0208 (6)	0.0164 (6)	0.000	0.0026 (5)	0.000
N3	0.0186 (6)	0.0259 (6)	0.0160 (6)	0.000	0.0010 (5)	0.000
C1	0.0176 (7)	0.0177 (7)	0.0205 (7)	0.000	0.0024 (6)	0.000
C2	0.0164 (7)	0.0220 (7)	0.0295 (8)	0.000	0.0036 (6)	0.000
C3	0.0216 (7)	0.0235 (7)	0.0338 (9)	0.000	0.0115 (6)	0.000
C4	0.0301 (8)	0.0229 (7)	0.0223 (8)	0.000	0.0100 (6)	0.000
C5	0.0225 (7)	0.0219 (7)	0.0201 (7)	0.000	0.0029 (6)	0.000
C6	0.0173 (7)	0.0176 (7)	0.0206 (7)	0.000	0.0031 (5)	0.000
C7	0.0150 (6)	0.0227 (7)	0.0146 (7)	0.000	0.0000 (5)	0.000
C8	0.0196 (5)	0.0265 (5)	0.0180 (5)	−0.0039 (4)	0.0002 (4)	−0.0018 (4)
C10	0.0155 (6)	0.0179 (7)	0.0179 (7)	0.000	−0.0014 (5)	0.000
C11	0.0167 (7)	0.0286 (8)	0.0217 (8)	0.000	−0.0038 (6)	0.000
C12	0.0171 (7)	0.0231 (7)	0.0156 (7)	0.000	−0.0017 (5)	0.000
C13	0.0176 (7)	0.0198 (7)	0.0184 (7)	0.000	0.0012 (5)	0.000
C14	0.0204 (7)	0.0281 (8)	0.0146 (7)	0.000	−0.0012 (5)	0.000
C15	0.0216 (7)	0.0289 (8)	0.0162 (7)	0.000	0.0035 (6)	0.000
C16	0.0188 (7)	0.0204 (7)	0.0189 (7)	0.000	0.0011 (5)	0.000
C17	0.0204 (7)	0.0227 (7)	0.0154 (7)	0.000	−0.0019 (5)	0.000
C18	0.0216 (7)	0.0255 (7)	0.0152 (7)	0.000	0.0026 (6)	0.000
C19	0.0203 (7)	0.0216 (7)	0.0218 (8)	0.000	0.0018 (6)	0.000
C20	0.0187 (7)	0.0527 (11)	0.0234 (8)	0.000	0.0037 (6)	0.000

### Geometric parameters (Å, º)

**Table:**

O1—C19	1.2163 (19)	C8—H8A	0.9800
N1—C10	1.3580 (18)	C8—H8B	0.9800
N1—C1	1.4084 (19)	C8—H8C	0.9800
N1—C11	1.4541 (19)	C10—C12	1.373 (2)
N2—N3	1.2868 (18)	C11—H11A	0.983 (15)
N2—C12	1.3567 (18)	C11—H11B	0.98 (2)
N3—C13	1.4101 (18)	C12—H12	0.9500
C1—C2	1.388 (2)	C13—C14	1.400 (2)
C1—C6	1.396 (2)	C13—C18	1.412 (2)
C2—C3	1.393 (2)	C14—C15	1.386 (2)
C2—H2	0.9500	C14—H14	0.9500
C3—C4	1.386 (2)	C15—C16	1.398 (2)
C3—H3	0.9500	C15—H15	0.9500
C4—C5	1.395 (2)	C16—C17	1.405 (2)
C4—H4	0.9500	C16—C19	1.494 (2)
C5—C6	1.381 (2)	C17—C18	1.378 (2)
C5—H5	0.9500	C17—H17	0.9500
C6—C7	1.5132 (19)	C18—H18	0.9500
C7—C10	1.5310 (19)	C19—C20	1.505 (2)
C7—C8^i^	1.5365 (13)	C20—H20A	0.95 (2)
C7—C8	1.5365 (13)	C20—H20B	0.983 (17)
			
C10—N1—C1	111.44 (12)	H8B—C8—H8C	109.5
C10—N1—C11	124.21 (13)	N1—C10—C12	123.59 (13)
C1—N1—C11	124.35 (12)	N1—C10—C7	109.11 (12)
N3—N2—C12	114.17 (12)	C12—C10—C7	127.30 (13)
N2—N3—C13	113.32 (12)	N1—C11—H11A	110.9 (8)
C2—C1—C6	122.29 (14)	N1—C11—H11B	109.8 (12)
C2—C1—N1	129.04 (14)	H11A—C11—H11B	109.6 (10)
C6—C1—N1	108.67 (12)	N2—C12—C10	118.86 (13)
C1—C2—C3	116.83 (14)	N2—C12—H12	120.6
C1—C2—H2	121.6	C10—C12—H12	120.6
C3—C2—H2	121.6	C14—C13—N3	115.60 (13)
C4—C3—C2	121.70 (14)	C14—C13—C18	118.97 (13)
C4—C3—H3	119.2	N3—C13—C18	125.43 (13)
C2—C3—H3	119.2	C15—C14—C13	120.59 (13)
C3—C4—C5	120.48 (14)	C15—C14—H14	119.7
C3—C4—H4	119.8	C13—C14—H14	119.7
C5—C4—H4	119.8	C14—C15—C16	120.81 (14)
C6—C5—C4	118.79 (14)	C14—C15—H15	119.6
C6—C5—H5	120.6	C16—C15—H15	119.6
C4—C5—H5	120.6	C15—C16—C17	118.28 (13)
C5—C6—C1	119.91 (13)	C15—C16—C19	122.40 (13)
C5—C6—C7	130.49 (13)	C17—C16—C19	119.33 (13)
C1—C6—C7	109.60 (13)	C18—C17—C16	121.56 (13)
C6—C7—C10	101.19 (11)	C18—C17—H17	119.2
C6—C7—C8^i^	111.23 (8)	C16—C17—H17	119.2
C10—C7—C8^i^	111.41 (8)	C17—C18—C13	119.79 (14)
C6—C7—C8	111.24 (8)	C17—C18—H18	120.1
C10—C7—C8	111.41 (8)	C13—C18—H18	120.1
C8^i^—C7—C8	110.12 (12)	O1—C19—C16	120.98 (14)
C7—C8—H8A	109.5	O1—C19—C20	120.33 (14)
C7—C8—H8B	109.5	C16—C19—C20	118.70 (13)
H8A—C8—H8B	109.5	C19—C20—H20A	111.7 (14)
C7—C8—H8C	109.5	C19—C20—H20B	111.3 (9)
H8A—C8—H8C	109.5	H20A—C20—H20B	108.5 (12)
			
C12—N2—N3—C13	180.0	C6—C7—C10—N1	0.000 (1)
C10—N1—C1—C2	180.000 (1)	C8^i^—C7—C10—N1	118.30 (8)
C11—N1—C1—C2	0.000 (1)	C8—C7—C10—N1	−118.30 (8)
C10—N1—C1—C6	0.000 (1)	C6—C7—C10—C12	180.000 (1)
C11—N1—C1—C6	180.000 (1)	C8^i^—C7—C10—C12	−61.70 (8)
C6—C1—C2—C3	0.000 (1)	C8—C7—C10—C12	61.70 (8)
N1—C1—C2—C3	180.000 (1)	N3—N2—C12—C10	180.000 (1)
C1—C2—C3—C4	0.000 (1)	N1—C10—C12—N2	180.000 (1)
C2—C3—C4—C5	0.000 (1)	C7—C10—C12—N2	0.000 (1)
C3—C4—C5—C6	0.000 (1)	N2—N3—C13—C14	180.0
C4—C5—C6—C1	0.000 (1)	N2—N3—C13—C18	0.000 (1)
C4—C5—C6—C7	180.000 (1)	N3—C13—C14—C15	180.0
C2—C1—C6—C5	0.000 (1)	C18—C13—C14—C15	0.000 (1)
N1—C1—C6—C5	180.000 (1)	C13—C14—C15—C16	0.0
C2—C1—C6—C7	180.000 (1)	C14—C15—C16—C17	0.000 (1)
N1—C1—C6—C7	0.000 (1)	C14—C15—C16—C19	180.0
C5—C6—C7—C10	180.000 (1)	C15—C16—C17—C18	0.000 (1)
C1—C6—C7—C10	0.000 (1)	C19—C16—C17—C18	180.000 (1)
C5—C6—C7—C8^i^	61.57 (8)	C16—C17—C18—C13	0.000 (1)
C1—C6—C7—C8^i^	−118.43 (8)	C14—C13—C18—C17	0.000 (1)
C5—C6—C7—C8	−61.58 (8)	N3—C13—C18—C17	180.000 (1)
C1—C6—C7—C8	118.42 (8)	C15—C16—C19—O1	180.0
C1—N1—C10—C12	180.000 (1)	C17—C16—C19—O1	0.000 (1)
C11—N1—C10—C12	0.000 (1)	C15—C16—C19—C20	0.000 (1)
C1—N1—C10—C7	0.000 (1)	C17—C16—C19—C20	180.0
C11—N1—C10—C7	180.000 (1)		

Symmetry code: (i) *x*, −*y*, *z*.

### Hydrogen-bond geometry (Å, º)

**Table:**

*D*—H···*A*	*D*—H	H···*A*	*D*···*A*	*D*—H···*A*
C2—H2···O1^ii^	0.95	2.57	3.5227 (19)	179
C14—H14···N3^iii^	0.95	2.55	3.4985 (19)	175
C11—H11*A*···*Cg*3^iv^	0.981 (15)	2.665 (14)	3.5230 (4)	145.8 (11)

Symmetry codes: (ii) *x*−1, *y*, *z*; (iii) −*x*+1, *y*, −*z*; (iv) −*x*−1, −*y*, −*z*.
